# Prevalence of ESBL-Producing *Enterobacter* Species Resistant to Carbapenems in Iran: A Systematic Review and Meta-Analysis

**DOI:** 10.1155/2022/8367365

**Published:** 2022-10-21

**Authors:** Farzad Khademi, Hamid Vaez, Zohreh Neyestani, Amirhossein Sahebkar

**Affiliations:** ^1^Department of Microbiology, School of Medicine, Ardabil University of Medical Sciences, Ardabil, Iran; ^2^Department of Microbiology, School of Medicine, Zabol University of Medical Sciences, Zabol, Iran; ^3^Biotechnology Research Center, Pharmaceutical Technology Institute, Mashhad University of Medical Sciences, Mashhad, Iran; ^4^Applied Biomedical Research Center, Mashhad University of Medical Sciences, Mashhad, Iran; ^5^Department of Biotechnology, School of Pharmacy, Mashhad University of Medical Sciences, Mashhad, Iran

## Abstract

**Background:**

Carbapenems are the last-line therapy for multidrug-resistant (MDR) infections caused by *Enterobacterales,* including those caused by *Enterobacter* species. However, the recent emergence of carbapenem-resistant (CR) and extended-spectrum *β*-lactamase (ESBL)-producing *Enterobacteriaceae* pathogens, which are resistant to nearly all antibiotics, has raised concerns among international healthcare organizations. Hence, because there is no comprehensive data in Iran, the current study aimed to evaluate the prevalence of antibiotic resistance among *Enterobacter* species, especially CR and ESBL-producing strains, in Iran.

**Methods:**

The literature search was performed up to June 21, 2021, in national and international databases using MeSH-extracted keywords, i.e., *Enterobacter*, antibiotic resistance, carbapenem, ESBL, and Iran. Study selection was done based on the predefined inclusion and exclusion criteria, and data analysis was carried out using the Comprehensive Meta-Analysis (CMA) software.

**Results:**

The pooled prevalence of *Enterobacter* species resistant to various antibiotics is as follows: imipenem 16.6%, meropenem 16.2%, aztreonam 40.9%, ciprofloxacin 35.3%, norfloxacin 31%, levofloxacin 48%, gentamicin 42.1%, amikacin 30.3%, tobramycin 37.2%, tetracycline 50.1%, chloramphenicol 25.7%, trimethoprim/sulfamethoxazole 52%, nalidixic acid 49.1%, nitrofurantoin 43%, ceftriaxone 49.3%, cefixime 52.4%, cefotaxime 52.7%, ceftazidime 47.9%, cefepime 43.6%, and ceftizoxime 45.5%. The prevalence rates of MDR and ESBL-producing *Enterobacter* species in Iran were 63.1% and 32.8%, respectively.

**Conclusion:**

In accordance with the warning of international organizations, our results revealed a high prevalence of ESBL-producing *Enterobacter* species in Iran, which is probably associated with the high prevalence of *Enterobacter* species resistant to most of the assessed antibiotics, especially MDR strains. However, the resistance rate to carbapenems was relatively low, and these drugs can still be considered as drugs of choice for the treatment of *Enterobacter* infections in Iran. Nevertheless, continuous monitoring of drug resistance along with antibiotic therapy based on the local data and evaluation of the therapeutic efficacy of new antibiotics or combination therapeutic strategies, such as ceftazidime/avibactam, meropenem/vaborbactam, plazomicin, and eravacycline, is recommended.

## 1. Introduction

The genus *Enterobacter* includes three medically important species, i.e., *Enterobacter cloacae* complex, *Enterobacter aerogenes* complex, and *Enterobacter sakazakii* [1, 2]. These enteric Gram-negative rods belong to the *Enterobacteriaceae* family and rarely cause infection in immunocompetent patients, but they are commonly associated with nosocomial infections, especially by the *Enterobacter cloacae* complex, in neonates and immunocompromised patients [1–6]. The most common nosocomial infections associated with these lactose-fermenting *Enterobacter* species include pneumonia, urinary tract infection, septicemia, and wound infection, as well as device-associated infections [1, 2]. Like many bacterial infections, in which an increasing trend of antibiotic resistance has led to the emergence of public health problems and imposed economic costs on healthcare, such an increasing trend of antibiotic resistance has also been reported for *Enterobacter* species [3, 6]. Among different mechanisms of resistance to various antibiotics in these Gram-negative rods, the intrinsic or acquired production of antibiotic-inactivating enzymes such as *β*-lactamases is very important [1]. *Enterobacter* species producing AmpC chromosomal cephalosporins are intrinsically resistant to ampicillin as well as first- and second-generation cephalosporins [2]. Plasmid-encoded extended-spectrum *β*-lactamase (ESBL) genes are involved in *Enterobacter* species' resistance to most *β*-lactam antibiotics, including second- and third-generation cephalosporins and aztreonam [6]. On the other hand, acquired resistance to quinolones, aminoglycosides, and carbapenems has been identified in hospital-acquired strains, which is highly important because these antibiotics are the last line of treatment [2, 4].

Recently, based on the World Health Organization (WHO) report, CR and ESBL-producing *Enterobacteriaceae* have been identified as one of the greatest threats to human health [5]. Although *Escherichia* and *Klebsiella* species are two main threats among CR and ESBL-producing *Enterobacteriaceae* [3], in the United States, CR *Enterobacter* species are considered the second most common CR *Enterobacteriaceae* [6].

However, there is no comprehensive data on antibiotic resistance patterns of *Enterobacter* species, especially CR strains, and ESBL-mediated resistance mechanisms in Iran. Therefore, the current systematic review and meta-analysis were designed to determine the prevalence of antibiotic resistance patterns of *Enterobacter* species, especially carbapenem-resistant strains, along with the frequency of ESBL-producing strains in Iran.

## 2. Methods

### 2.1. Literature Search and Study Selection

International databases including PubMed, Scopus, and Google Scholar, along with national databases including Scientific Information Database (https://www.sid.ir/) and Magiran (https://www.magiran.com/), were searched independently by two investigators to find studies conducted on the prevalence of antibiotic resistance and ESBL-producing *Enterobacter* species in Iran. The search was performed from 1996 to June 21, 2021. The most common Medical Subject Headings (MeSH)-extracted keywords used for the literature search were as follows: *Enterobacter*, antibiotic resistance, carbapenem, ESBL, and Iran. We defined the inclusion and exclusion criteria for the studies retrieved in the search and selected studies that met our criteria after a review of the titles, abstracts, and full text of the articles. The following studies were removed from the meta-analysis: studies reporting antibiotic resistance and ESBL-positive isolates published in languages other than English or Persian, studies conducted in other countries, studies reporting other bacteria in the *Enterobacteriaceae* family, studies with a small sample size (less than 10 bacterial isolates), studies with insufficient data, and nonoriginal articles, abstracts, and duplicates. Reference lists of the included articles were checked in order to find any possible missed studies. The current systematic review and meta-analysis were designed according to the PRISMA (Preferred Reporting Items for Systematic Reviews and Meta-Analysis) guidelines [7].

### 2.2. Data Extraction

Two different investigators extracted the data, and a third investigator tabulated the required information in Table 1 after resolving possible disagreements in the results of the search and reaching a consensus. Required data were as follows: first author's surname, study location, study enrollment date, the number of isolates, antibiotic susceptibility testing methods, the prevalence of *Enterobacter* species resistance to different drugs, the prevalence of multidrug-resistant (MDR) *Enterobacter* species, and the frequency of ESBL-positive isolates. It is noteworthy that *Enterobacter* species have intrinsic resistance to *β*-lactam antibiotics including ampicillin, amoxicillin-clavulanate, ampicillin-sulbactam, cephalosporins I (cefazolin and cephalothin), cephamycins (cefoxitin and cefotetan), and cephalosporin II (cefuroxime). According to the Clinical and Laboratory Standards Institute (CLSI) guideline, susceptibility testing is unnecessary for the above-mentioned antibiotics [8]. For this reason, these antibiotics are not included in Table 1.

### 2.3. Data Analysis

In the current study, Cochrane's *Q* test (chi-squared, *χ*^2^) and Higgins *I*^2^ statistics were used to assess heterogeneity across the included studies. For this purpose, if the *p* value was less than 0.1 for the *χ*^2^ test and the *I*^2^ value was higher than 25%, the presence of heterogeneity was considered and a random-effects model was applied for the meta-analysis. Extracted data on the prevalence of *Enterobacter* species' antibiotic resistance and ESBL-producing species in Iran were expressed as a percentage and 95% confidence intervals (95% CIs). Additionally, a subgroup analysis was performed based on the location of the study. A funnel plot-based method was used for reporting the presence or absence of publication bias in the meta-analyses, and it was considered a potential sign of publication bias if the graph showed an asymmetric shape. The Comprehensive Meta-Analysis (CMA) software (Biostat, Englewood, NJ) was used for the meta-analysis.

## 3. Results

**Figure 1:**
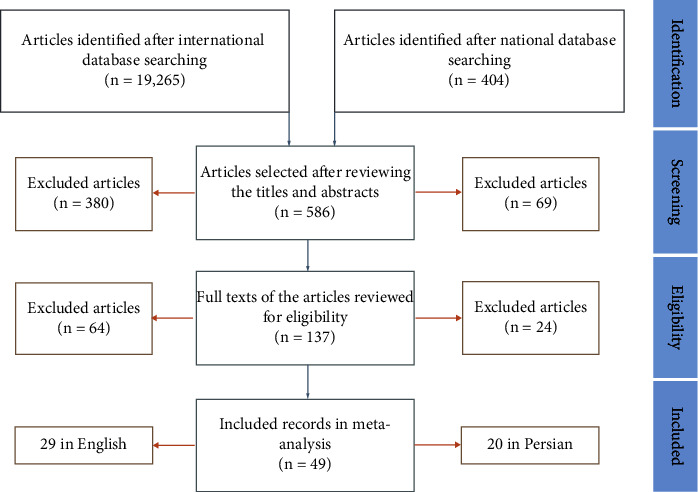
A schematic view of the study selection process.

**Figure 2:**
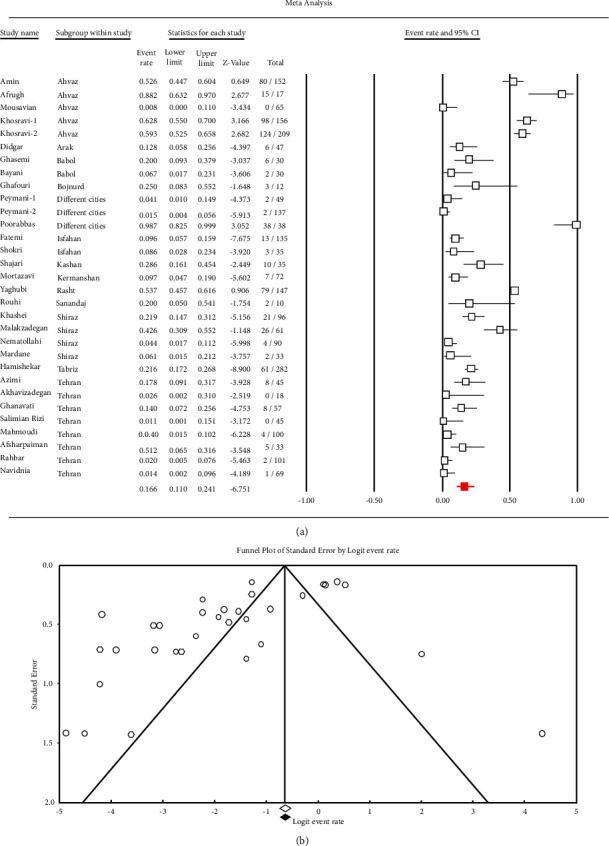
Forest plots (a) and funnel plots (b) illustrate the prevalence of imipenem-resistant *Enterobacter* species in Iran.

Among 19,669 eligible studies published from 1996 until June 21, 2021, 49 articles (20 in Persian and 29 in English) met the inclusion criteria and were included in the meta-analysis (Figure 1). As shown in Table 1, data were obtained from 19 cities (Ahvaz (*n* = 5), Arak (*n* = 1), Babol (*n* = 2), Bojnurd (*n* = 1), Fasa (*n* = 1), Hamadan (*n* = 1), Ilam (*n* = 1), Isfahan (*n* = 2), Jahrom (*n* = 1), Kashan (*n* = 1), Kerman (*n* = 1), Kermanshah (*n* = 2), Rasht (*n* = 2), Sanandaj (*n* = 4), Semnan (*n* = 1), Shiraz (*n* = 4), Tabriz (*n* = 2), Tehran (*n* = 13), and Zahedan (*n* = 1)) in Iran. All studies used the disk diffusion method for antimicrobial susceptibility testing. The pooled prevalence of *Enterobacter* species' resistance to various antibiotics was as follows: imipenem 16.6% (95% *CI*: 11–24.1; *I*^2^ = 93.1%; *Q* = 439.9; *p* ≤ 0.001) (Figure 2), meropenem 16.2% (95% *CI*: 8.9–27.9; *I*^2^ = 89.8%; *Q* = 117.8; *p* ≤ 0.001), aztreonam 40.9% (95% *CI*: 29.6–53.2; *I*^2^ = 89.3%; *Q* = 75; *p* ≤ 0.001), ciprofloxacin 35.3% (95% *CI*: 29.5–41.6; *I*^2^ = 86.1%; *Q* = 273.6; *p* ≤ 0.001), norfloxacin 31% (95% *CI*: 14.3–54.7; *I*^2^ = 91.6%; *Q* = 59.9; *p* ≤ 0.001), levofloxacin 48% (95% *CI*: 21.3–75.9; *I*^2^ = 90.7%; *Q* = 32.4; *p* ≤ 0.001), gentamicin 42.1% (95% *CI*: 36.2–48.3; *I*^2^ = 87.2%; *Q* = 328.5; *p* ≤ 0.001), amikacin 30.3% (95% *CI*: 24.5–36.8; *I*^2^ = 86.9%; *Q* = 298.8; *p* ≤ 0.001), tobramycin 37.2% (95% *CI*: 26.3–49.5; *I*^2^ = 88.3%; *Q* = 103.1; *p* ≤ 0.001), tetracycline 50.1% (95% *CI*: 37.3–62.9; *I*^2^ = 88%; *Q* = 134; *p* ≤ 0.001), chloramphenicol 25.7% (95% *CI*: 20.5–31.6; *I*^2^ = 61.1%; *Q* = 20.5; *p* ≤ 0.001), trimethoprim/sulfamethoxazole 52% (95% *CI*: 45.4–58.6; *I*^2^ = 87.5%; *Q* = 304.9; *p* ≤ 0.001), nalidixic acid 49.1% (95% *CI*: 38.8–59.4; *I*^2^ = 87.6%; *Q* = 177.4; *p* ≤ 0.001), nitrofurantoin 43% (95% *CI*: 32.4–54.2; *I*^2^ = 91.7%; *Q* = 328.8; *p* ≤ 0.001), ceftriaxone 49.3% (95% *CI*: 41.8–56.9; *I*^2^ = 87.1%; *Q* = 226.1; *p* ≤ 0.001), cefixime 52.4% (95% *CI*: 43.7–61; *I*^2^ = 83.4%; *Q* = 102.5; *p* ≤ 0.001), cefotaxime 52.7% (95% *CI*: 42.4–62.7; *I*^2^ = 91.9%; *Q* = 359.3; *p* ≤ 0.001), ceftazidime 47.9% (95% *CI*: 39.8–56.2; *I*^2^ = 89.7%; *Q* = 302; *p* ≤ 0.001), cefepime 43.6% (95% *CI*: 31.3–56.8; *I*^2^ = 90.1%; *Q* = 142.2; *p* ≤ 0.001) and ceftizoxime 45.5% (95% *CI*: 30.6–61.3; *I*^2^ = 92.7%; *Q* = 178.4; *p* ≤ 0.001).

In addition, Table 2 shows the antibiotic resistance profiles of *Enterobacter* species in different cities of Iran. The rate of MDR *Enterobacter* species in Iran was 63.1% (95% *CI*: 45.2–78; *I*^2^ = 93.9%; *Q* = 249.1; *p* ≤ 0.001).

In addition, the prevalence of ESBL-producing *Enterobacter* species was 32.8% (95% *CI*: 23.3–44; *I*^2^ = 79.4%; *Q* = 29.1; *p* ≤ 0.001) in Iran.

It should be noted that a random-effects model was applied for the meta-analysis due to the existence of high heterogeneity across the included studies in this study.

## 4. Discussion

The emergence of MDR- and ESBL-producing *Enterobacteriaceae*, including *Enterobacter* species, has increased the necessity to deal with these organisms [5, 6]. The Centers for Disease Control and Prevention (CDC) estimated 197,400 cases of ESBL-producing *Enterobacteriaceae* along with 9,100 deaths among hospitalized patients in the United States in 2017 [58]. The antibiotic of choice to treat infections caused by MDR and ESBL-producing *Enterobacteriaceae* is carbapenem [3, 58, 59]. However, the widespread use of carbapenem antibiotics has led to the emergence of CR bacteria [3, 59]. According to the CDC report for 2019, increased prevalence of CR *Enterobacteriaceae*, especially CR *Enterobacter cloacae* complex, has become a public health issue in the United States [58].

In Iran, the prevalence of MDR (63.1%) and ESBL-producing *Enterobacter* species (32.8%) was high. This is an alarming rate despite the relatively low frequency of imipenem- and meropenem-resistant *Enterobacter* species in Iran. The results suggest that carbapenems are still the drugs of choice for the treatment of infections caused by MDR and ESBL-producing *Enterobacter* species in Iran. The distribution of ESBL-producing *Enterobacter* species in other countries was as follows: Pakistan 14.9%, Nigeria 37.5%, and Ethiopia 50% [60, 61].

The CDC has reported that CR *Enterobacteriaceae*-associated infections frequently occur in patients using medical devices, including catheters (intravenous and urinary) and ventilators, and some of these microorganisms are resistant to all available antibiotics, hence their infections are difficult to treat [58]. Currently, the available antimicrobial agents for the treatment of CR *Enterobacteriaceae* are limited [62]. Historically, aminoglycosides, tigecycline, polymyxins, and fosfomycin have been used as therapeutic options for this purpose [62]. However, according to the included articles in this study, there is insufficient data on the prevalence of tigecycline-, polymyxins-, and fosfomycin-resistant *Enterobacter* species in Iran. Hence, the evaluation of *Enterobacter* species resistance rates to these antibiotics is recommended. In the present study, the rate of tetracycline-resistant *Enterobacter* species was high (50.1%).

On the other hand, aminoglycosides, including gentamicin, amikacin, and tobramycin, are also recommended as anti-CR *Enterobacteriaceae* therapies [62]. However, based on the present study, the prevalence of gentamicin-, amikacin-, and tobramycin-resistant *Enterobacter* species was high in Iran. It is recommended that older antibiotics such as trimethoprim/sulfamethoxazole and chloramphenicol may be effective for the treatment of infections caused by CR *Enterobacteriaceae* pathogens [62]. Our results showed that the prevalence of *Enterobacter* species resistant to chloramphenicol was higher than those resistant to trimethoprim/sulfamethoxazole (25.7% vs. 52%). Other treatment options for infections caused by CR *Enterobacteriaceae* include combination strategies (high-dose tigecycline, high-dose carbapenem, and double-carbapenem therapy), new antibiotics (ceftazidime/avibactam, meropenem/vaborbactam, plazomicin, and eravacycline), and new antibiotics in development (imipenem/cilastatin, relebactam, and cefiderocol) [62]. However, information on the therapeutic efficacy of these drugs against CR *Enterobacter* species is not available in Iran (according to the included articles in this study). Based on the current study, the frequency of meropenem and ceftazidime-resistant *Enterobacter* species was 16.2% and 47.9%, respectively. *Enterobacter* species' drug resistance rates to the third-generation cephalosporins and aztreonam were high in Iran. Considering the prevalence of ESBL-producing *Enterobacter* species in this study (32.8%), it seems that these ESBLs are involved in resistance to third-generation cephalosporins and aztreonam in Iran. The CDC estimated the rate of quinolone-resistant *Enterobacter* species as 30% [3]; however, the prevalence of *Enterobacter* species resistant to quinolones was higher in this study.

Such a high antibiotic resistance of *Enterobacter* species, especially MDR, in this study can be attributed to the indiscriminate use of antibiotics and easy, without a prescription, access to antibiotics and self-medication in Iran [63, 64]. On the other hand, since *Enterobacter* species are responsible for nosocomial infections, using appropriate infection control programs and practices of hygiene such as hand decontamination, glove use, sterilization, and disinfection practices can play an important role in preventing the spread of resistant strains in healthcare settings.

One of the limitations of the current study was the inability to compare the obtained results with other countries, particularly adjacent countries, which needs to be addressed in future multicenter and international studies.

## 5. Conclusion

This study is the first systematic review and meta-analysis reporting *Enterobacter* species antibiotic resistance in Iran. The results of this meta-analysis indicated the high prevalence of *Enterobacter* species resistant to the majority of assessed antibiotics in the included studies, i.e., quinolones, aminoglycosides, third- and fourth-generation cephalosporins, aztreonam, tetracycline, chloramphenicol, trimethoprim/sulfamethoxazole, and nitrofurantoin. In addition, the prevalence rates of ESBL-producing *Enterobacter* species (32.8%) and MDR (63.1%) strains were high in Iran. Such an increasing trend of antibiotic resistance in *Enterobacter* species can impose more economic costs on healthcare systems in Iran due to prolonged periods of hospitalization, increased drug consumption, poor patient outcomes, and higher mortality and morbidity. In total, we suggest the management of antibiotic prescription, launching and developing health education and infection control programs, continuous monitoring of drug resistance, and evaluation of the therapeutic efficacy of new antimicrobial agents (herbal medicine and new antimicrobial peptides) or combination therapeutic strategies are required to control *Enterobacter* species-associated infections and antibiotic resistance in Iran. Finally, in comparison with the above-mentioned antibiotics, the prevalence of CR *Enterobacter* species was relatively low in Iran, and it seems that carbapenems can still be considered as drugs of choice for the treatment of MDR and ESBL-producing *Enterobacter* species.

## Figures and Tables

**Table 1:** Required data were extracted from included articles in the meta-analysis.

Author (Ref)	City	Year	Isolate (*n*)	AST	Resistance rate (*n*)																					ESBL-positive (n)
					IPM	MEM	ATM	CIP	NOR	LVX	GEN	AMK	TOB	TET	CHL	SXT	NAL	NIT	CRO	CFM	CTX	CAZ	CEP	ZOX	MDR	
Amin et al. [9]	Ahvaz	2015-2016	152	Disk diffusion	80	53	ND	84	70	63	ND	ND	ND	ND	ND	ND	ND	ND	ND	ND	ND	ND	ND	ND	ND	ND
Afrugh et al. [10]	Ahvaz	2013-2014	17	Disk diffusion	15	15	ND	15	ND	ND	14	16	ND	17	ND	12	16	13	16	ND	17	15	14	16	ND	ND
Mousavian et al. [11]	Ahvaz	2012	65	Disk diffusion	0	ND	ND	6	ND	ND	5	3	ND	ND	ND	ND	ND	ND	10	ND	11	9	ND	11	ND	27
Khosravi et al. [12]	Ahvaz	2009–2012	156	Disk diffusion	98	ND	ND	91	ND	ND	102	60	ND	64	ND	108	94	75	121	110	ND	ND	ND	ND	ND	ND
Khosravi et al. [13]	Ahvaz	2009-2010	209	Disk diffusion	124	ND	ND	88	ND	ND	143	117	ND	124	ND	146	119	117	143	148	ND	ND	ND	ND	ND	ND
Didgar [14]	Arak	2010–2012	47	Disk diffusion	6	ND	ND	19	ND	ND	19	18	ND	ND	ND	20	ND	ND	29	ND	ND	40	25	19	ND	ND
Ghasemi et al. [15]	Babol	2020	30	Disk diffusion	6	ND	ND	6	ND	ND	4	6	ND	ND	ND	ND	ND	9	ND	ND	10	ND	28	ND	28	ND
Bayani et al. [16]	Babol	2011-2012	30	Disk diffusion	2	ND	ND	2	ND	ND	ND	2	ND	ND	ND	ND	ND	ND	ND	ND	ND	5	4	ND	0	ND
Ghafouri et al. [17]	Bojnurd	2013	12	Disk diffusion	3	5	ND	8	ND	ND	8	2	ND	3	0	6	ND	1	6	0	0	1	ND	ND	ND	ND
Peymani et al. [18]	Different cities	2014	49	Disk diffusion	2	2	27	16	ND	ND	20	11	ND	ND	ND	25	ND	ND	28	ND	34	27	ND	ND	26	ND
Peymani et al. [19]	Different cities	2011-2012	137	Disk diffusion	2	1	67	22	16	ND	59	ND	ND	ND	ND	83	ND	ND	78	ND	80	71	ND	ND	83	ND
Poorabbas et al. [20]	Different cities	2008-2009	38	Disk diffusion	38	ND	ND	33	ND	35	24	26	0	ND	ND	22	ND	22	18	13	19	20	24	ND	ND	ND
Molazade et al. [21]	Fasa	2012-2013	28	Disk diffusion	ND	ND	ND	11	ND	ND	8	0	ND	11	ND	14	11	11	11	11	0	ND	ND	ND	ND	ND
Esmaeili et al. [22]	Hamadan	2011	15	Disk diffusion	ND	ND	ND	13	ND	ND	13	ND	13	ND	ND	10	6	4	11	ND	ND	ND	ND	ND	ND	ND
Yasemi et al. [23]	Ilam	2007–2009	20	Disk diffusion	ND	ND	ND	ND	ND	ND	3	ND	ND	ND	ND	9	ND	6	ND	ND	ND	ND	ND	ND	ND	ND
Fatemi et al. [24]	Isfahan	2014-2015	135	Disk diffusion	13	16	93	58	ND	ND	54	46	53	96	26	23	ND	ND	ND	ND	89	87	90	ND	98	ND
Shokri et al. [25]	Isfahan	2012-2013	35	Disk diffusion	3	3	ND	21	ND	ND	22	10	ND	ND	ND	ND	ND	6	ND	ND	24	22	12	ND	35	ND
Kargar et al. [26]	Jahrom	2011-2012	25	Disk diffusion	ND	ND	ND	ND	ND	ND	17	7	11	24	ND	20	24	ND	ND	ND	10	10	ND	ND	ND	1
Shajari et al. [27]	Kashan	2005-2006	35	Disk diffusion	10	ND	ND	13	ND	ND	14	12	11	ND	ND	21	28	ND	ND	18	22	22	ND	ND	ND	ND
Sepehri et al. [28]	Kerman	1996, 2000	72	Disk diffusion	ND	ND	ND	ND	ND	ND	43	ND	ND	ND	ND	46	12	28	ND	ND	ND	ND	ND	ND	ND	ND
Mortazavi et al. [29]	Kermanshah	2016-2017	72	Disk diffusion	7	ND	29	35	30	ND	36	35	ND	ND	ND	49	31	16	ND	40	37	38	ND	ND	54	ND
Amini et al. [30]	Kermanshah	2015	18	Disk diffusion	ND	ND	ND	7	ND	ND	8	9	ND	ND	ND	13	7	5	6	6	ND	10	ND	ND	ND	ND
Karambin and Zarkesh [31]	Rasht	2008–2010	50	Disk diffusion	ND	ND	ND	0	ND	ND	15	41	ND	ND	ND	40	ND	ND	ND	ND	43	ND	ND	ND	ND	ND
Yaghubi et al. [32]	Rasht	2013–2015	147	Disk diffusion	79	61	ND	80	ND	ND	81	66	78	0	ND	108	105	102	102	119	109	92	ND	80	ND	ND
Rouhi et al. [33]	Sanandaj	2013-2014	10	Disk diffusion	2	ND	ND	2	0	ND	0	5	ND	2	ND	ND	0	0	3	ND	5	4	ND	5	ND	ND
Nikkhoo et al. [34]	Sanandaj	2009-2010	11	Disk diffusion	ND	ND	ND	3	ND	ND	7	6	ND	7	ND	2	ND	ND	6	5	5	ND	ND	ND	ND	ND
Ramazanzadeh et al. [35]	Sanandaj	2007-2008	28	Disk diffusion	ND	ND	ND	14	22	ND	3	6	ND	ND	ND	13	23	ND	9	ND	7	7	ND	5	ND	ND
Afkhamzadeh et al. [36]	Sanandaj	2007-2008	15	Disk diffusion	ND	ND	ND	10	ND	ND	11	7	ND	10	ND	10	ND	ND	13	ND	13	4	ND	ND	ND	ND
Jazayeri [37]	Semnan	1999	11	Disk diffusion	ND	ND	ND	5	ND	ND	5	ND	ND	ND	ND	10	10	10	ND	8	ND	ND	ND	ND	ND	ND
Khashei et al. [38]	Shiraz	2016-2017	96	Disk diffusion	21	ND	ND	31	ND	ND	39	22	ND	ND	ND	45	ND	68	ND	ND	ND	70	ND	ND	93	35
Malekzadegan et al. [39]	Shiraz	2015-2016	61	Disk diffusion	26	ND	ND	30	ND	ND	46	61	ND	ND	ND	21	3	4	ND	ND	ND	55	ND	ND	56	ND
Nematolahi et al. [40]	Shiraz	2005–2014	90	Disk diffusion	4	1	44	14	ND	ND	33	27	ND	ND	30	ND	ND	ND	52	62	55	47	34	ND	10	14
Mardaneh et al. [41]	Shiraz	2013	33	Disk diffusion	2	7	14	5	ND	ND	11	7	14	24	8	9	ND	ND	14	19	15	13	13	ND	ND	13
Rezaee and Abdinia [42]	Tabriz	2010–2014	40	Disk diffusion	ND	ND	ND	0	ND	ND	20	0	ND	ND	ND	40	40	40	20	ND	40	ND	ND	10	40	ND
Hamishehkar et al. [43]	Tabriz	2010–2012	282	Disk diffusion	61	ND	ND	89	ND	ND	84	61	ND	ND	78	109	ND	25	79	ND	41	55	ND	49	ND	ND
Azimi et al. [44]	Tehran	2013–2018	45	Disk diffusion	8	23	11	9	ND	7	26	15	36	45	ND	20	9	45	30	ND	38	31	32	39	12	ND
Akhavizadegan et al. [45]	Tehran	2016-2017	18	Disk diffusion	0	ND	ND	7	ND	7	ND	0	ND	ND	ND	8	6	2	5	7	ND	4	2	ND	ND	ND
Sohrabi fard et al. [46]	Tehran	2014-2015	12	Disk diffusion	ND	ND	ND	ND	ND	ND	ND	ND	ND	4	ND	ND	ND	ND	5	ND	ND	ND	ND	ND	ND	ND
Ghanavati et al. [47]	Tehran	2013-2014	57	Disk diffusion	8	ND	ND	ND	ND	ND	ND	ND	ND	ND	ND	ND	ND	ND	ND	ND	28	21	ND	ND	10	30
Salimian rizi et al. [48]	Tehran	2012-2013	45	Disk diffusion	0	ND	14	6	ND	ND	10	6	10	26	8	28	ND	ND	ND	ND	13	15	7	ND	45	ND
Mahmoudi et al. [49]	Tehran	2011–2016	100	Disk diffusion	4	ND	ND	ND	ND	ND	27	30	ND	ND	ND	20	ND	ND	ND	ND	69	ND	36	ND	46	ND
Rajabi et al. [50]	Tehran	2011–2012	17	Disk diffusion	ND	ND	ND	2	ND	ND	ND	ND	ND	2	ND	2	ND	9	ND	ND	ND	ND	ND	ND	ND	ND
Afsharpaiman et al. [51]	Tehran	2011	33	Disk diffusion	5	0	ND	ND	ND	ND	17	14	ND	ND	16	18	11	30	14	14	30	22	ND	9	ND	ND
Rahbar et al. [52]	Tehran	2010-2011	101	Disk diffusion	2	ND	14	ND	ND	ND	15	3	19	19	19	20	ND	ND	ND	ND	20	23	5	ND	ND	33
Ranjbar et al. [53]	Tehran	2006-2007	83	Disk diffusion	ND	ND	ND	32	ND	ND	41	49	44	ND	22	47	47	60	45	50	ND	54	ND	53	ND	ND
Taheri et al. [54]	Tehran	2004–2012	14	Disk diffusion	ND	ND	ND	ND	0	ND	9	4	ND	ND	ND	6	1	5	2	ND	ND	0	ND	2	ND	ND
Haghi et al. [55]	Tehran	2003-2004	39	Disk diffusion	ND	ND	ND	ND	ND	ND	13	5	11	ND	ND	17	23	23	13	16	ND	16	ND	12	ND	ND
Navidinia et al. [56]	Tehran	NA	69	Disk diffusion	1	1	ND	12	ND	ND	5	2	4	ND	ND	ND	ND	ND	39	ND	ND	ND	43	67	4	ND
Sadeghi bojd et al. [57]	Zahedan	2013–2015	32	Disk diffusion	ND	ND	ND	3	ND	ND	2	3	ND	ND	ND	14	9	2	6	6	6	ND	ND	ND	ND	ND

IPM-imipenem; MEM-meropenem; ATM-aztreonam; CIP-ciprofloxacin; NOR-norfloxacin; LVX-levofloxacin; GEN-gentamicin; AMK-amikacin; TOB-tobramycin; TET-tetracycline; CHL-chloramphenicol; SXT-trimethoprim/sulfamethoxazole; NAL-nalidixic acid; NIT-nitrofurantoin; CRO-ceftriaxone; CFM-cefixime; CTX-cefotaxime; CAZ-ceftazidime; CEP-cefepime; ZOX-ceftizoxime; MDR-multidrug-resistant; ESBL-extended-spectrum *β*-lactamase; AST-antimicrobial susceptibility testing; ND-not determined.

**Table 2:** Antibiotic resistance profile of *Enterobacter* isolates from cities in Iran.

City	Percentage resistance (%)																			
	IPM	MEM	ATM	CIP	NOR	LVX	GEN	AMK	TOB	TET	CHL	SXT	NAL	NIT	CRO	CFM	CTX	CAZ	CEP	ZOX
Ahvaz	58	64.4	NA	46.8	46.1	41.4	53.1	40.9	NA	56.7	NA	69.6	61.2	55.1	63.6	70.7	68.8	50.9	82.4	61.8
Arak	12.8	NA	NA	40.4	NA	NA	40.4	38.3	NA	NA	NA	42.6	NA	NA	61.7	NA	NA	85.1	53.2	40.4
Babol	13.2	NA	NA	13.2	NA	NA	13.3	13.2	NA	NA	NA	NA	NA	30	NA	NA	33.3	16.7	58.8	NA
Bojnurd	25	41.7	NA	66.7	NA	NA	66.7	16.7	NA	25	3.8	50	NA	8.3	50	3.8	3.8	8.3	NA	NA
Fasa	NA	NA	NA	39.3	NA	NA	28.6	1.7	NA	39.3	NA	50	39.3	39.3	39.3	39.3	1.7	NA	NA	NA
Hamadan	NA	NA	NA	86.7	NA	NA	86.7	NA	86.7	NA	NA	66.7	40	26.7	73.3	NA	NA	NA	NA	NA
Ilam	NA	NA	NA	NA	NA	NA	15	NA	NA	NA	NA	45	NA	30	NA	NA	NA	NA	NA	NA
Isfahan	9.4	11.3	68.9	49.9	NA	NA	50.3	33	39.3	71.1	19.3	17	NA	17.1	NA	NA	66.5	64.1	51.4	NA
Jahrom	NA	NA	NA	NA	NA	NA	68	28	44	96	NA	80	96	NA	NA	NA	40	40	NA	NA
Kashan	28.6	NA	NA	37.1	NA	NA	40	34.3	31.4	NA	NA	60	80	NA	NA	51.4	62.9	62.9	NA	NA
Kerman	NA	NA	NA	NA	NA	NA	59.7	NA	NA	NA	NA	63.9	16.7	38.9	NA	NA	NA	NA	NA	NA
Kermanshah	9.7	NA	40.3	46.7	41.7	NA	48.9	48.9	NA	NA	NA	68.9	42.2	23.4	33.3	46.8	51.4	53.3	NA	NA
Rasht	53.7	41.5	NA	11.8	NA	NA	42.8	65.1	53.1	0.3	NA	75	71.4	69.4	69.4	81	79.4	62.6	NA	54.4
Sanandaj	20	NA	NA	43.2	34.2	NA	33.5	40.5	NA	51.6	NA	45.2	36.4	4.5	50.7	45.5	51.2	28.6	NA	30.7
Semnan	NA	NA	NA	45.5	NA	NA	45.5	NA	NA	NA	NA	90.9	90.9	90.9	NA	72.7	NA	NA	NA	NA
Shiraz	15.1	5.9	47.2	26.9	NA	NA	46.8	34.7	42.4	72.7	31.1	37.5	4.9	29.9	51.7	65.1	54.8	66.5	38.2	NA
Tabriz	21.6	NA	NA	9.1	NA	NA	38.3	7.5	NA	NA	27.7	85.3	98.8	70.8	37.4	NA	75.9	19.5	NA	19
Tehran	6.3	6.7	22	22.9	3.3	25	33.1	18.9	31.2	42.8	26.4	39.4	36.3	63.1	45.3	47.6	58.6	41.8	28.8	59.2
Zahedan	NA	NA	NA	9.4	NA	NA	6.3	9.4	NA	NA	NA	43.8	28.1	6.3	18.8	18.8	18.8	NA	NA	NA

NA-not available.

## Data Availability

No data were used to support this study.
